# Molecular dynamics simulations data of the twenty encoded amino acids in different force fields

**DOI:** 10.1016/j.dib.2016.02.086

**Published:** 2016-03-09

**Authors:** F. Vitalini, F. Noé, B.G. Keller

**Affiliations:** a Department of Biology, Chemistry, Pharmacy, Freie Universität Berlin, Takustraße 3, D-14195 Berlin, Germany; b Department of Mathematics and Computer Science, Freie Universität Berlin, Arnimallee 6, D-14195 Berlin, Germany

**Keywords:** Molecular dynamics, Force field, Amino acid

## Abstract

We present extensive all-atom Molecular Dynamics (MD) simulation data of the twenty encoded amino acids in explicit water, simulated with different force fields. The termini of the amino acids have been capped to ensure that the dynamics of the *Φ* and ψ torsion angles are analogues to the dynamics within a peptide chain. We use representatives of each of the four major force field families: AMBER ff-99SBILDN [1], AMBER ff-03 [2], OPLS-AA/L [3], CHARMM27 [4] and GROMOS43a1 [5], [6]. Our data represents a library and test bed for method development for MD simulations and for force fields development. Part of the data set has been previously used for comparison of the dynamic properties of force fields (Vitalini et al., 2015) [7] and for the construction of peptide basis functions for the variational approach to molecular kinetics [8].

**Specifications Table**

**Table:** 

Subject area	*Chemistry, Biology*
More specific subject area	*Computational Molecular biophysics*
Type of data	*Molecular Dynamics (MD) simulations*
How data was acquired	Classical all-atom MD simulation in explicit solvent
Data format	GROMACS [9] trajectory output xtc
Experimental factors	NVT ensemble at 300 K
Experimental features	GROMACS 4.5.5 [9] software
Data source location	Freie Universität Berlin Germany
Data accessibility	Data within this article

**Value of the data**

• The dataset constitutes a library of extensive all-atom simulations of the basic dynamic building block of peptides and proteins. The total simulation time in the data set amounts to 200 μs.
• MD simulations represent a powerful tool to investigate the time evolution of biomolecules at atomistic resolution. For small systems, such as amino acids, it represents the only technique to probe the structural details of the dynamic processes.
• New methods for the analysis of MD simulations are often tested on alanine dipeptide (Ac-A-NHMe), for which many groups have trajectories available. Our data set extends the set of available test cases to all twenty encoded amino acids.
• The data can be used to construct peptide basis functions for the variational approach to molecular kinetics [10], [11], as described in Ref. [8].
• Our data set allows the user to probe and compare the properties of the force fields [7] and to make an informed decision when choosing a force field for the simulation of larger systems.

## 1 Data

The public repository (ftp://bdg.chemie.fu-berlin.de/Ac-X-NHMe/) is structured as following. In the main folder a README.txt file can be found. It illustrates the simulation details common to all the set-ups, also described in Experimental Design, Material and Methods A. A GROMACS-specific [9] simulation parameters file (nvt_production_1mus.mdp) is also adduced for clarity.

The data is sorted according to force field. In each force-field subfolder twenty folders are present, one per amino acid. The folders are denoted as Ac-X-NHMe, where X is replaced with the one letter code of the amino acid. Within an amino-acid specific folder, another README.txt summarizes the number and length of the independent runs. A sub-folder is associated to each independent run, including the initial configuration Ac-X-NHMe_run0.gro and the trajectory in (GROMACS binary) format (.xtc). Moreover, a topology file (Ac-X-NHMe.top) is given, which contains the atom types, the bonded and non-bonded parameters of the force field of choice, and lists the constraints. The initial configuration (.gro), the topology file (.top) and the simulation file (.mdp) permit the re-run of the simulations.

## 2 Experimental design, materials and methods

### 2.1 MD simulations

We performed all-atom MD simulations in explicit solvent of terminally blocked amino acids, acetyl-X-methylamide (Ac-X-NHMe), where X stands for any of the twenty encoded amino acids. All twenty amino acids were simulated with five different force fields: AMBER ff-99SB-ILDN [1], AMBER ff-03 [2], OPLS-AA/L [3], CHARMM27 [4] and GROMOS43a1 [5], [6]. The water model was chosen to be in agreement with the one used for the validation of the force field, i.e. TIP3P [12] for AMBER ff99SB-ILDN, AMBER ff03, OPLS-AA/L and CHARMM27, and SPC [13] for GROMOS43a1. Simulations were performed with the GROMACS 4.5.5 simulation package9. The number of particles and the volume were fixed during the simulations. Temperature was restrained at 300 K using the V-Rescale thermostat [14]. Each initial set up was minimised using the steepest descent algorithm and equilibrated in the NVT ensemble for 100 ps. Subsequently two independent production runs of 1 μs each, were carried out for each amino acid/force field combination (exception: aliphatic amino acids A, G, I, L and P in ff99SB-ILDN [1] force field, production runs of 200 ns each). This yields to a total simulation time of 2 μs per simulation setup (exception: aliphatic amino acids A, G, I, L and P in ff99SB-ILDN [1] force field, 4 μs; A, V in ff-03 [2], OPLS-AA/L [3], CHARMM27 [4] and GROMOS43a1 [5], [6], 4 μs). The integration time-step was of 2 fs and atom positions of the solute were written to file every 1 ps. In the production runs, the leap-frog intergrator was used and bonds to hydrogen atoms were constrained using the LINCS algorithm [15] (lincs iter=1, lincs order=4). A cut-off of 1 nm was used for Lennard–Jones interactions. Electrostatic interactions were treated by the Particle-Mesh Ewald (PME) algorithm [16] in combination with a real space cut-off of 1 nm, a grid spacing of 0.16 nm, and an interpolation order of 4. Periodic boundary conditions were applied in all three dimensions. For further details refer to Table 1.

### 2.2 Ramachandran plots

**Fig. 1:**
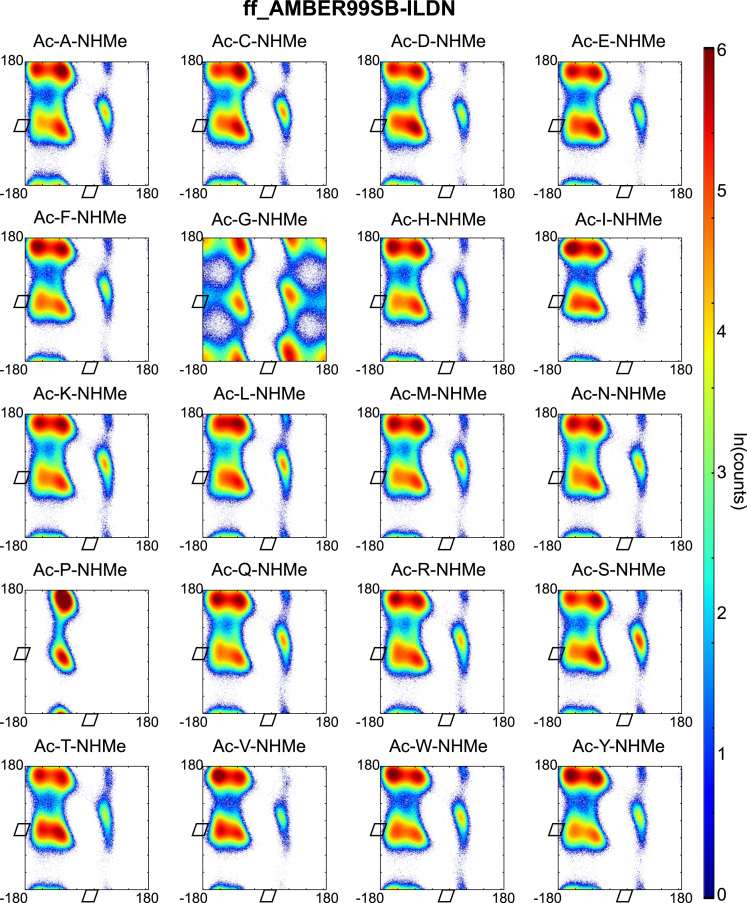
Ramachandran plots of all terminally capped amino acids simulated with AMBER ff99SB-ILDN force field. Represented is the logarithm of the {*Φ*-*ψ*}-pairs counts on a 1°-grid.

**Fig. 2:**
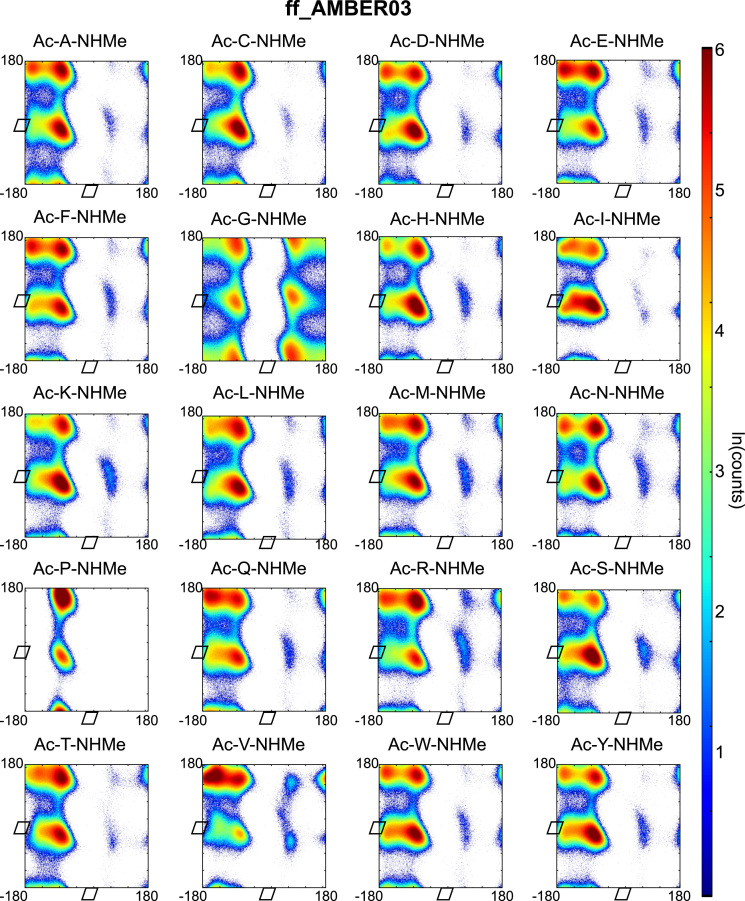
Ramachandran plots of all terminally capped amino acids simulated with AMBER ff03 force field. Represented is the logarithm of the {*Φ*-*ψ*}-pairs counts on a 1°-grid.

**Fig. 3:**
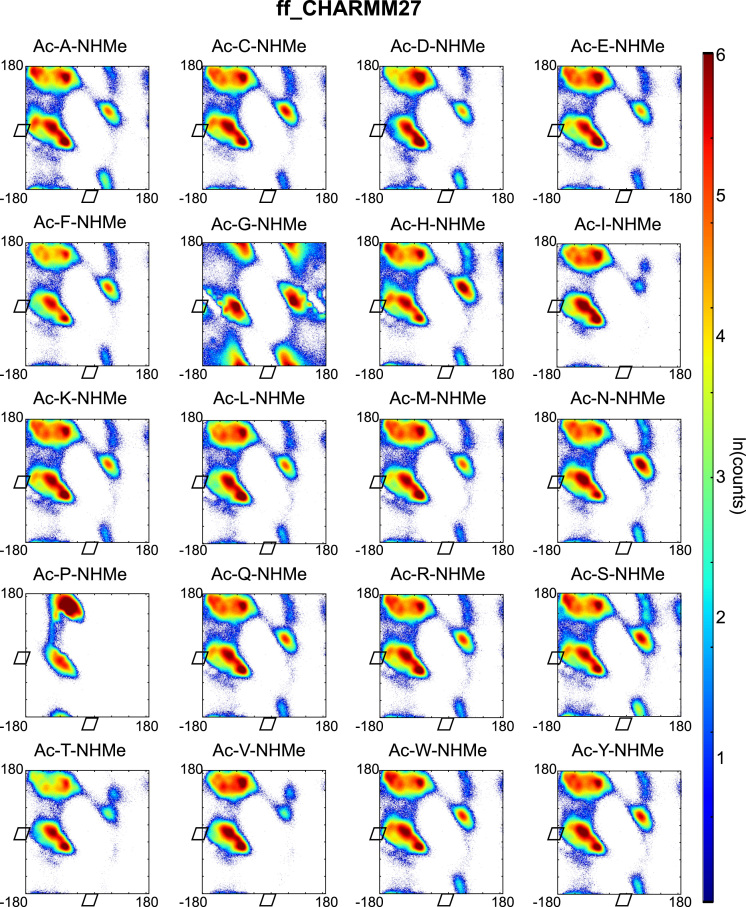
Ramachandran plots of all terminally capped amino acids simulated with CHARMM27 force field. Represented is the logarithm of the {*Φ*-*ψ*}-pairs counts on a 1°-grid.

**Fig. 4:**
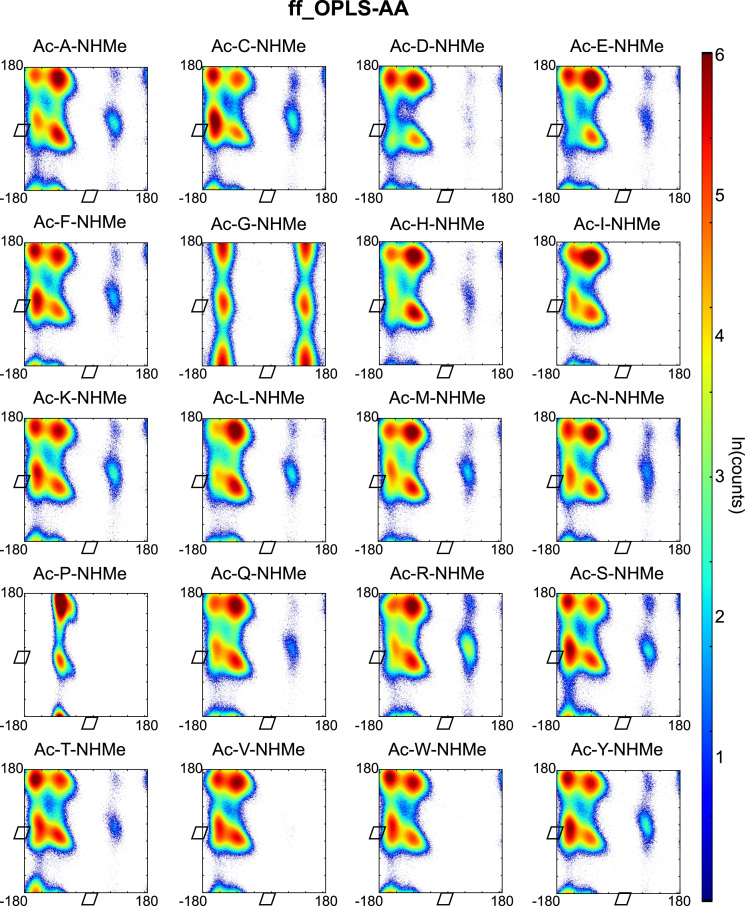
Ramachandran plots of all terminally capped amino acids simulated with OPLS-AA/L force field. Represented is the logarithm of the {*Φ*-*ψ*}-pairs counts on a 1°-grid.

**Fig. 5:**
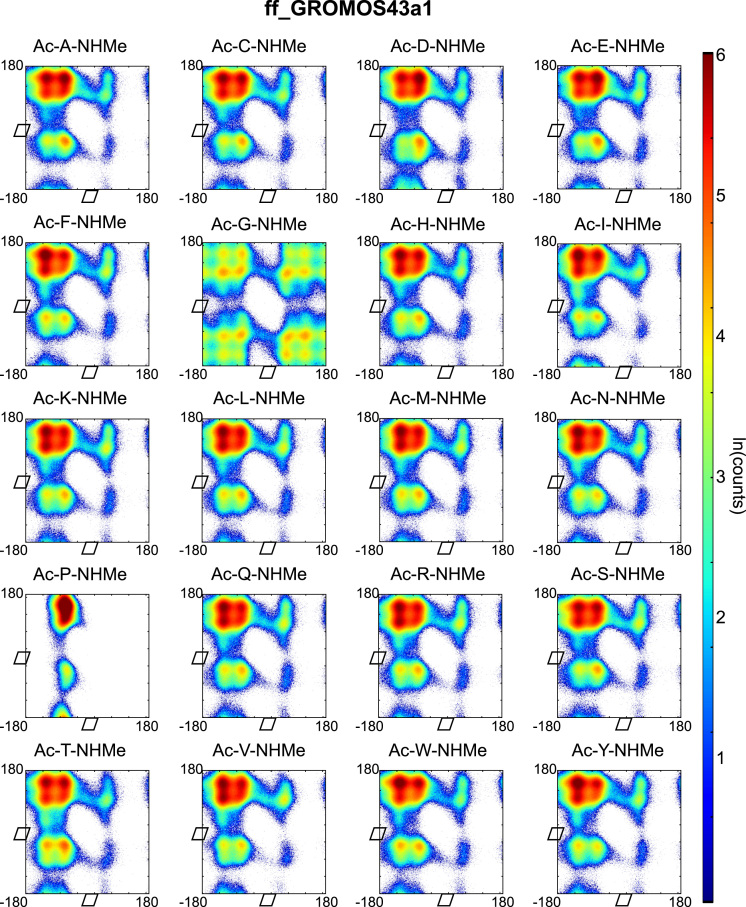
Ramachandran plots of all terminally capped amino acids simulated with GROMOS43a1 force field. Represented is the logarithm of the {*Φ*-*ψ*}-pairs counts on a 1°-grid.

Backbone dihedral angles *Φ* and ψ are good reaction coordinates for the dynamics of amino acids and short peptides. Using the GROMACS command g_rama we extracted the *Φ* and *ψ* time-series from the MD trajectories. The space spanned by the {*Φ−ψ*}-combinations of a single amino acid (capped or within a peptide-chain), has a well-defined distribution and can be represented in a two-dimensional plot known as Ramachandran plot. We constructed a Ramachandran plot for each of the amino acid-force field combinations by making an histogram of the *Φ* and *ψ* time-series over 2 μs of simulation time onto a regular grid of 360×360 bins (bin-width 1° per coordinate). The natural logarithm of such histogram counts are shown in Fig. 1, Fig. 2, Fig. 3, Fig. 4, Fig. 5 as indication of the exploration of the configurational space over the simulation time.

## Figures and Tables

**Table 1:** Simulation parameters per amino acid and force field: number of water molecules, size of simulation box, number of independent runs and total simulation time.

Amino Acid	ff_AMBER ff99SB -ILDN			ff_AMBER ff03			ff_CHARMM27			ff_OPLS AA/L			ff_GROMOS43a1		
# H2O	box-size	sim. time	# H2O	box-size	sim. time	# H2O	box-size	sim. time	# H2O	box-size	sim. time	# H2O	box-size	sim. time
Ac-A-NHMe	651	(2.7 nm)^3^	20×200 ns	646	(2.7 nm) ^3^	4×1 μs	680	(2.8 nm) ^3^	4×1 μs	684	(2.8 nm) ^3^	4×1 μs	531	(2.5 nm) ^3^	4×1 μs
Ac-C-NHMe	717	(2.8 nm) ^3^	2×1 μs	717	(2.8 nm) ^3^	2×1 μs	717	(2.8 nm) ^3^	2×1 μs	717	(2.8 nm) ^3^	2×1 μs	610	(2.7 nm) ^3^	2×1 μs
Ac-D-NHMe	763	(2.9 nm) ^3^	2×1 μs	763	(2.9 nm) ^3^	2×1 μs	751	(2.8 nm) ^3^	2×1 μs	763	(2.9 nm) ^3^	2×1 μs	648	(2.7 nm) ^3^	2×1 μs
Ac-E-NHMe	796	(2.9 nm) ^3^	2×1 μs	796	(2.9 nm) ^3^	2×1 μs	796	(2.9 nm) ^3^	2×1 μs	796	(2.9 nm) ^3^	2×1 μs	749	(2.8 nm) ^3^	2×1 μs
Ac-F-NHMe	903	(3.0 nm) ^3^	2×1 μs	903	(3.0 nm) ^3^	2×1 μs	911	(3.0 nm) ^3^	2×1 μs	903	(3.0 nm) ^3^	2×1 μs	854	(3.0 nm) ^3^	2×1 μs
Ac-G-NHMe	795	(2.9 nm) ^3^	20×200 ns	643	(2.7 nm) ^3^	3×1 μs	628	(2.7 nm) ^3^	2×1 μs	644	(2.7 nm) ^3^	2×1 μs	534	(2.5 nm) ^3^	2×1 μs
Ac-H-NHMe	848	(2.9 nm) ^3^	2×1 μs	848	(2.9 nm) ^3^	2×1 μs	854	(2.9 nm) ^3^	2×1 μs	848	(2.9 nm) ^3^	2×1 μs	785	(2.9 nm) ^3^	2×1 μs
Ac-I-NHMe	766	(2.9 nm) ^3^	20×200 ns	751	(2.9 nm) ^3^	4×1 μs	751	(2.8 nm) ^3^	2×1 μs	751	(2.8 nm) ^3^	2×1 μs	636	(2.7 nm) ^3^	2×1 μs
Ac-K-NHMe	924	(3.0 nm) ^3^	2×1 μs	924	(3.0 nm) ^3^	2×1 μs	924	(3.0 nm) ^3^	2×1 μs	924	(3.0 nm) ^3^	2×1 μs	876	(3.0 nm) ^3^	2×1 μs
Ac-L-NHMe	796	(2.9 nm) ^3^	20×200 ns	726	(2.8 nm) ^3^	4×1 μs	739	(2.8 nm) ^3^	2×1 μs	726	(2.8 nm) ^3^	2×1 μs	622	(2.7 nm) ^3^	2×1 μs
Ac-M-NHMe	782	(2.9 nm) ^3^	2×1 μs	782	(2.9 nm) ^3^	2×1 μs	782	(2.9 nm) ^3^	2×1 μs	782	(2.9 nm) ^3^	2×1 μs	664	(2.7 nm) ^3^	2×1 μs
Ac-N-NHMe	722	(2.8 nm) ^3^	2×1 μs	722	(2.8 nm) ^3^	2×1 μs	727	(2.8 nm) ^3^	2×1 μs	722	(2.8 nm) ^3^	2×1 μs	679	(2.8 nm) ^3^	2×1 μs
Ac-P-NHMe	860	(3.0 nm) ^3^	20×200 ns	704	(2.8 nm) ^3^	4×1 μs	723	(2.8 nm) ^3^	2×1 μs	704	(2.8 nm) ^3^	2×1 μs	610	(2.7 nm) ^3^	2×1 μs
Ac-Q-NHMe	881	(3.0 nm) ^3^	2×1 μs	881	(3.0 nm) ^3^	2×1 μs	881	(3.0 nm) ^3^	2×1 μs	881	(3.0 nm) ^3^	2×1 μs	824	(2.9 nm) ^3^	2×1 μs
Ac-R-NHMe	1026	(3.2 nm) ^3^	2×1 μs	1026	(3.2 nm) ^3^	2×1 μs	1026	(3.2 nm) ^3^	2×1 μs	1026	(3.2 nm) ^3^	2×1 μs	997	(3.1 nm) ^3^	2×1 μs
Ac-S-NHMe	691	(2.8 nm) ^3^	2×1 μs	691	(2.8 nm) ^3^	2×1 μs	706	(2.8 nm) ^3^	2×1 μs	691	(2.8 nm) ^3^	2×1 μs	614	(2.7 nm) ^3^	2×1 μs
Ac-T-NHMe	748	(2.8 nm) ^3^	2×1 μs	748	(2.8 nm) ^3^	2×1 μs	724	(2.8 nm) ^3^	2×1 μs	748	(2.8 nm) ^3^	2×1 μs	626	(2.7 nm) ^3^	2×1 μs
Ac-V-NHMe	676	(2.8 nm) ^3^	20×200 ns	672	(2.8 nm) ^3^	4×1 μs	672	(2.8 nm) ^3^	4×1 μs	672	(2.8 nm) ^3^	4×1 μs	577	(2.6 nm) ^3^	4×1 μs
Ac-W-NHMe	930	(3.0 nm) ^3^	2×1 μs	930	(3.0 nm) ^3^	2×1 μs	916	(3.0 nm) ^3^	2×1 μs	930	(3.0 nm) ^3^	2×1 μs	869	(3.0 nm) ^3^	2×1 μs
Ac-Y-NHMe	99-	(3.1 nm) ^3^	2×1 μs	990	(3.1 nm) ^3^	2×1 μs	990	(3.1 nm) ^3^	2×1 μs	990	(3.1 nm) ^3^	2×1 μs	925	(3.0 nm) ^3^	2×1 μs
