# Supplementary material for: Molecular dynamics simulations data of the twenty encoded amino acids in different force fields
Source: Data Brief. 2016 Mar 9;7:582–90. doi: 10.1016/j.dib.2016.02.086 (PMC4802541; doi:10.1016/j.dib.2016.02.086)

Conflicts of Interest Statement

The authors of the manuscript “Molecular dynamics simulation data of the twenty encoded amino acids in different force fields”, submitted to the Data in Brief Journal with ID DIB-D-15-00583R1, certify that they have no conflict of interest in the subject discussed in the manuscript.

Zürich, 16/2/2016

Francesca Vitalini

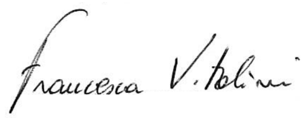

Supplement: Supplementary file 1 — Supplementary material [file mmc1.pdf]
